# Possible Imprinting and Microchimerism in Chronic Lymphocytic Leukemia and Related Lymphoproliferative Disorders

**Published:** 2008-02-10

**Authors:** Viggo Jønsson, Geir E. Tjønnfjord, Tom B. Johannesen, Sven Ove Samuelsen, Bernt Ly

**Affiliations:** 1 Department of Hematology, Aker University Hospital, University of Oslo, Norway; 2 Department of Hematology, Rikshospital and Radium Hospital, University of Oslo, Norway; 3,5 The National Norwegian Cancer Registry, Oslo, Norway; 4 Department of Biostatistics, Faculty of Mathematics, University of Oslo, Norway

**Keywords:** chronic lymphocytic leukaemia, imprinting, microchimerism

## Abstract

Based on the concept that the tumorogenesis in chronic lymphocytic leukaemia comprises both an initial, inherited mutation and subsequent somatic mutations, the pleiotypic diversity of familial chronic lymphocytic leukaemia and related malignant lymphoproliferative disorders is generally explained by a repertoire of monoallelic polygenes in the initial mutation. Epigenetic genomic imprinting is a likely mechanism behind of the asynchroneous replicating monoallelic polygenes which is discussed in the light of pleiotrophy and birth order effect. Furthermore, it is discussed that one possible mechanism available for the epigenetic transfer of these genes could be the physiological pregnancy-related microchimerism between mother and fetus.

It is generally accepted that chronic lymphocytic leukemia (CLL) is a monoclonal B-lymphoproliferative disorder caused by a mutation in the differentiation pathway of the B-lymphocyte and that this mutation causes an arrest in the differentiation with accumulation of the leukemic subset of immature monoclonal B-lymphocytes. Since these B-lymphocytes are genuinely circulating in blood and lymph, the leukemic cells are spread to all part of the body with vessels and accumulated especially in bone marrow, lymph nodes and spleen and sometimes even in lamina propria and in other extra-lymphoid tissues [1–5]. Related monoclonal lymphocytic disorders (LDs) such as the other chronic leukemias (Waldenström’s macroglobulinemia, hairy cell leukemia, prolymphocytic leukemia and lymphoma cell leukemia), non-Hodgkin lymphomas, Hodgkin’s disease and multiple myeloma are defined from their specific mutated arrests in the differentiation and amplification [2,6].

The multistep evolution of LD is based on a paradigm (Figs. 1 and 2) comprising two different types of mutations (i) an initial mutation most likely under the influence of genetic factors, and (ii) subsequent somatic mutations which to our knowledge today are not convincingly genetically disposed. The purpose of the present paper is to discuss the influence of imprinting and microchimerism in the genetics of CLL and related LDs.

## Evidence of Heredity

A hereditary mechanism in CLL and LDs is seen from the marked familial clustering with pleiotrophy, which means familial co-expression of CLL and LDs [7–17]. The hereditary momentum of these disorders is furthermore reflected by the fact that one major risk factor of CLL is the presence of another family member with CLL and/or another type of LD and that CLL ethnically is mainly seen among people from the Western world [18].

**Figure 1:**
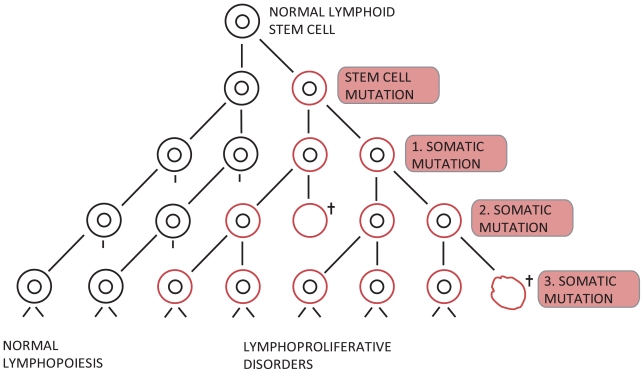
Pathogenesis of the lymphoproliferative disorders. The model is based on the assumption that an initial, inherited stem cell mutation with subsequent somatic mutations cause pleiotypic segregation of the different diagnoses within the entity of LPD, for example the acute and chronic lymphocytic leukemias, malignant lymphomas including Hodgkin’s disease and multiple myeloma.

**Figure 2:**
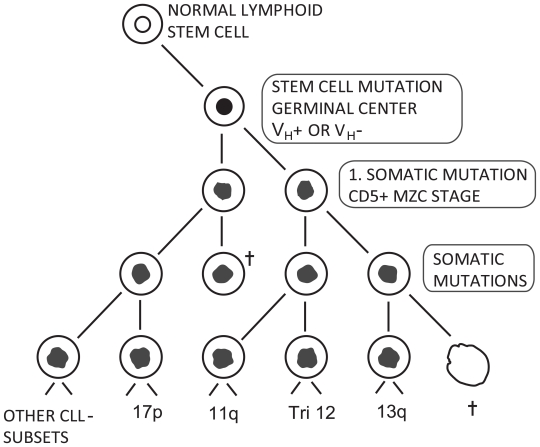
Pathogenesis of chronic lymphocytic leukemia (CLL). By definition, CLL is a mutated monoclone of B-lymphocytes with the expression of CD5, CD19, CD20, CD23 and weak SmIg. This monoclone has a growth-pattern different from normal lymphocytes and is divided into two subtypes with or without variable gene H rearrangement for coding of immunoglobulin production (the VH mutation) with good and poor prognosis, respectively. By means of FISH technique, both VH^+^ and VH^−^ CLL are further segregated into subsets, characterized by deletion 17p, deletion 11q, trisomy 12, deletion 6p, deletion 13q and other subtypes including one subtype with a normal FISH investigation. These subgroups constitute entities with different prognosis and need of treatment. After the genetically transmission of the primary stem cell mutation, the differentiation of the mutated B lymphocyte goes through a number of stages, first a marginal-zone(MZC)-like naïve-B-CD5+ stage, from where many or perhaps most cell line die, while del 17p, del 11q, tri 12, del 13q etc. survive via amplification in the abnormal stroma of the lymphoid tissue of CLL with marked cytokine stimulation and marked autoimmune micro-regulation. These genetic phenotypes with selective advantage in the CLL stroma [5] do not reflect the so far unknown genuine inherited DNA-alteration of the first somatic mutation.

However, to date the gene or genes involved have not been identified. A considerable number of publications on gene-defects in CLL address the subsequent somatic mutations (Fig. 1). There is no proof for the otherwise common assumption that the diagnostic FISH aberrations and VH profiles of the subsets of CLL are the primary and inheritable chromosomal defects. On the contrary, they are rather “phenotypes” for survival pathways of the subsets through the tumorogenesis (Fig. 2), sometimes unspecific to such an extent that they hardly can act as candidates to a primarily genetic defects. As for example the Philadelphia defect t(9; 22) which is demonstrable both in chronic myelogenous leukemia, acute lymphoblastic leukemia and in some types on non-Hodgkin’s lymphomas [19].

Neither linking studies [20–27] nor genome-wide screening [for review see 28] have exposed this or these genes related to the initial mutations. Their mode of transfer from generation to generation in affected families remains uncertain. A number of large-scaled computerized estimations of data from cancer registries confirm the pleiotrophic clustering and point out CLL as the subset of clustered LDs with the highest frequency. Thus, CLL is the most common diagnosis within LD having a stronger relative risk (RR) between two family members with CLL than between two family members with other combinations of LDs in some of the studies published, but so far, there are no hints to a possible Mendelian pattern of the genetic segregation. A remarkable fluctuation in the calculated RR within the entity of LD from different registries is reported from these studies [7–12]. The problems with such large-scaled registrations are changed definitions of disease with a new taxonomic system for LDs nearly every 10th year (Gall and Mallory, 1942; Rappaport, 1956; Good and Finstad, 1967, the Kieler Classification 1972, Working Formulation 1982 Real Classification 1994) up to the present WHO Formulation from 2001 [ 2 ] including redefinition of established disorders based on molecular-biological criteria, and the presence of low-grade conditions without clinical symptoms as for example MGUS (mono-clonal gammopathy of uncertain significance) and CLUS (chronic lymphocytosis of uncertain significance) with unknown incidences which, after all, make it highly difficult to compare LDs-data on incidences and frequencies from the past centuries covering the past generations in epidemiological studies. The power of these association studies can be enhanced using selected cases with a family history of LDs. Such genealogical studies (segregation analysis) of pedigrees do neither point out a specific pattern of genetic mechanism apart from the general finding that a genetic mode is undoubtedly on question [29].

Whether the somatic mutations in the development of LDs (Figs. 1 and 2) also are influenced by genetic factors is a matter for further discussion. Antigenic drive from for example Helicobacter pylori [30], HIV, EBV and hepatitis C virus [31,32] are well known stimulators for the development of LDs and thus, the genetic disposition to acquire and express such infections and the possible birth order effect caused by chronic contamination from older sibs have been brought into attention [33–36]. A birth order effect denotes an unequal and non-random occurrence of affected offspring in the sibship where the rank of affected and unaffected sibs is not as would be expected from a simple Mendelian segregation. A birth order effect can be due to non-genetic mechanism such as environmental infections or to parents’ decision after having had an affected offspring but certainly, a birth order effect can also be due to a genetic effect as a consequence of a pseudo-Hardy Weinberg formulation [37–39].

## Imprinting and Pregnancy Related Microchimerism

One of the major recent contributions to the discussion on familial CLL is the demonstration of a likely monoallelic polygene model where each monoallelic gene confer a small relative risk which increases when the monoalleleic genes occur in multiplicative and/or additional combinations [28,40–43].

If the interacting monoallelic polygenes arises as a consequence of inherited and initial mutations they mimic an autosomal dominant syndrome in which the individual cells in the mutated clone become homozygous for a recessive neoplasma causing gene [40–43]. During the progression of tumor and under the influence of subsequent mitoses, carcinogenous hits and somatic mutations (Figs. 1 and 2), the initial mutation in only one allel of a tumor suppressor gene or a DNA repair gene will often cause a loss of the wild-type allel, if the cell survive these damages at all. The initial heritable gene or genes are often lost or mutated somatically in this multistep evolution of tumor [41–43].

However, in spite of multiple damages some mutated cell lines survive leading to “cancer”. This diversity of surviving, mutated clones caused by multiple monoallelic genes explains most likely the pleiotypic appearance of subsets of diagnoses in families with clustering of CLL and related LDs. Such asynchronous replicating monoallelic genes are often either genomic imprinted genes or genes subjected to X-chromosome inactivation [44]. While a sole X-chromosome inactivation hardly has support from data so far, genomic imprinting seems a likely mechanism [45] giving a reasonable explanation of the otherwise elusive dominant inheritance of the lymphoproliferative disorders with unexplained pleiotyphy and birth order effect.

Genomic imprinting is a non-Mendelian gene expression depending upon the parent that transmit it and where the mechanism which silences the maternal or the paternal copy is epigenetic, meaning outside the genes [37–39, 46–51]. This pregnancy-related growth-factor segregation, where the mother selects the load of paternally and maternally genes for her offspring is presumably a normal physiological mechanism. However, the maternal selection of monoallelic genes for her offspring is hardly left to sheer coincidence. Evidently, this master plan comprises a mode so that the mother can “remember the haplo-load” from her past and present male partners. In other words, the mother must be able to operate a mechanism which can remember the paternal genes and that precise mechanism could well be the pregnancy related microchimerism [52–54]. Imprinted genes are monoallelically expressed and regulated independently of spermiogenesis and oogenesis by allele-specific epigenetic modifiers (silencer) where DNA methylation and/or modifications of histones are well described mechanisms [55]. Related to the parent — offspring transmission of CLL and other subsets of LD, one possible mechanism available for the epigenetic DNA methylation could be the physiological pregnancy-related microchimerism between mother and fetus, based on the normal traffic of lymphocytes and monocytes across the feto-maternal (utero-placental) barrier [52–54]. In each pregnancy, fetal cells are transplanted lifelong into the mother so that the mother accumulates increased tolerance to non-self from the paternal half of the fetus in step with an increased number of pregnancies and an increased number of male partners [56–59].

The parent’s age and the birth-order effect were originally united parameters in Haldane and Smith’s test from 1947 [60,61] based on the simple assumption that if a genetic malformation in a child depends on the age of the father, the disease of the child is most likely due to mutation; if it depends on the age of the mother the impact is uncertain; and if “the number of previous children is the main factor, it probably acts through biochemical reactions between mother an child” [62]. In contemporary terms, the demonstration of a paternal birth-order could be equal to “the number of previous children as the main factor”. Such a genetic mediation of a growth-factor mosaic comprising maternal or paternal growth-factor enhancers or inhibitors with maternal imprinting, viz. maternal silence of paternal LD-genes, and a marked male predominance in CLL is perfectly in accordance with genomic imprinting., viz. a non-Mendelian gene expression depending upon the parent that transmit it and where the mechanism which silences the maternal or the paternal copy is epigenetic.

Genomic imprinting is in favour of adjustment of different maternal resources to her offspring to maximize maternal fitness [63]. Perhaps also to prevent paternal genetic predominance in those cases where the female has been mating with many highly selected males. Avian biology learned us extreme examples where the female under cross-mating by males even maintain the sub-species [64]. We can only speculate on the elusive monoallelic polygenes for the growth-factors involved in familial CLL and LDs and how they vanish in the multistep evolution of tumor under the influence of multiple somatic mutations. The identification of these genes and the interpretation of their impact on RR of subsets of LD must be based on insight into the mode they are transferred from generation to generation. In this process we want to point out the resemblance to genomic imprinting. Much of what is known today on the genetics of CLL and related LDs seem to match genomic imprinting where the likely memory-mechanism of the mother to recruit and select parental monoallelic genes for her offspring could well be her pregnancy related microchimerism so that genomic imprinting and pregnancy related microchimerism are linked together in an operative system.
